# Eosinophils and drugs for eosinophilia are associated with the risk of colorectal cancer: a Mendelian randomization study

**DOI:** 10.18632/aging.206081

**Published:** 2024-08-23

**Authors:** Yuan-Yuan Wang, Zhi-Han Jia, Qing-Jun Wang, Zhi-Tu Zhu

**Affiliations:** 1 Cancer Clinical Research Ward, The First Affiliated Hospital of Jinzhou Medical University, Jinzhou, China; 2 Department of Oncology, The First Affiliated Hospital of Jinzhou Medical University, Jinzhou, China; 3 Liaoning Provincial Key Laboratory of Clinical Oncology Metabonomics, Institute of Clinical Bioinformatics, Cancer Center of Jinzhou Medical University, The First Affiliated Hospital of Jinzhou Medical University, Jinzhou, China

**Keywords:** eosinophils, cancer risk, colorectal cancer, Mendelian randomization, pan-cancer

## Abstract

Eosinophils have the potential to exhibit both anti-tumor properties and tumor-promoting effects. However, the impact of eosinophil levels in the bloodstream on tumorigenesis risk remains inadequately explored. Furthermore, investigations regarding the association between drugs regulating eosinophils and cancer risk are currently absent. In this study, we conducted a Mendelian randomization (MR) analysis utilizing eosinophil count and eosinophil percentage as exposures. In both cohorts, a significant association was observed between eosinophil count and the risk of colorectal cancer and skin malignancies. However, upon conducting a sensitivity analysis, heterogeneity was detected specifically in relation to skin malignancies. Subsequent reverse Mendelian randomization analysis did not indicate any evidence of reverse causality. Furthermore, the multivariate Mendelian randomization analysis results suggested that eosinophils act as a mediating factor in reducing the risk of colorectal cancer and skin malignancies in individuals with asthma. And the use of drugs that modulate eosinophilia may increase the risk of colorectal cancer. It is evident that the statistical evidence supporting a negative correlation between eosinophils count and the susceptibility to colorectal cancer is particularly robust. And, it is plausible to suggest that pharmaceutical interventions aimed at modulating eosinophilia may potentially heighten the risk of colorectal cancer. Hence, it is imperative to exercise caution and remain mindful of the potential risk of colorectal cancer when employing these medications.

## INTRODUCTION

Eosinophils have been implicated in a range of diseases, including asthma, helminth parasitic infections, and cancer [1]. In the context of various cancers, eosinophils serve as effector cells within the tumor microenvironment, enhancing host antitumor responses. Additionally, eosinophils secrete soluble mediators that facilitate angiogenesis and matrix remodeling, thereby promoting tumor growth [2, 3]. Current research primarily concentrates on investigating the influence of eosinophils on tumor cells and their impact on patient prognosis and treatment efficacy. However, the extent to which the quantity of eosinophils in the bloodstream affects tumor susceptibility remains incompletely understood.

A retrospective study revealed a potential association between circulating eosinophils and a decreased risk of colorectal cancer [4]. Conversely, another study demonstrated that patients exhibiting a linear increase in peripheral eosinophils had a heightened risk of developing colorectal cancer [5]. Furthermore, an analysis conducted on Asian populations indicated that eosinophils may confer a protective effect against lung cancer [6]. However, it is worth noting that no comprehensive pan-cancer investigations have been conducted to explore the relationship between eosinophils count, eosinophil percentage, and the risk of various cancers. Additionally, the impact of eosinophil-modulating medications on cancer risk remains unexplored in existing studies.

Mendelian randomization (MR) is one of the emerging approaches to strengthen causal inference based on the instrumental variable (IV) method. This approach is advantageous as it mitigates the presence of confounding or reverse causality biases, owing to the random distribution of genetic variation during meiosis, which remains independent of environmental factors, disease onset, and disease progression [7, 8].

The primary objective of this study was to investigate the association between eosinophil count, eosinophil percentage, and the susceptibility to different types of cancers through the application of MR method. Additionally, the study aimed to evaluate the potential impact of eosinophil modulation therapy on the corresponding risk of tumor development.

Our study found an inverse relationship between eosinophil count and the risk of colorectal cancer and skin malignant. The utilization of drugs targeting IL-4, and IL-13, which regulate eosinophils, may potentially elevate the risk of colorectal cancer. Furthermore, through multivariate analysis, we identified eosinophil count as a major factor in reducing risk of colorectal cancer and skin malignancy in individuals with asthma.

## RESULTS

### MR analysis of eosinophil count and cancer risk based on FinnGen database

**Figure 1:**
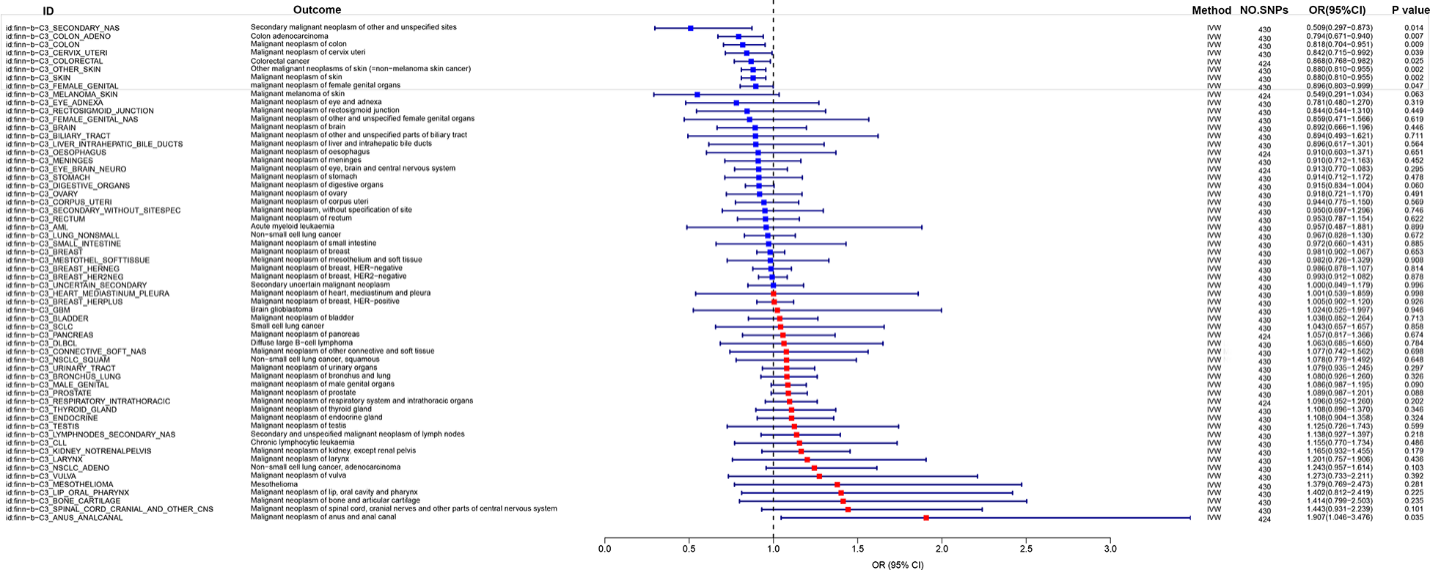
MR analysis of eosinophil count and cancer risk based on FinnGen database.

The present study aimed to investigate the correlation between eosinophil count and cancer risk by utilizing the eosinophil count with ID ieu-b-33 as the exposure data and examining 60 types of cancer in the FinnGen database as the outcome. The findings revealed an inverse relationship between eosinophil count and the risk of Other malignant neoplasms of skin (= non-melanoma skin cancer), Malignant neoplasm of skin, Colon adenocarcinoma, Malignant neoplasm of colon, and Secondary malignant neoplasm of other and unspecified sites, Colorectal cancer, Malignant neoplasm, Malignant neoplasm of cervix uteri, malignant neoplasm of female genital organs. And the P-values were all < 0.05. OR were all < 1, and the maximum value of 95% CI were all < 1 (Figure 1). Conversely, a positive association was observed between eosinophil count and the risk of Malignant neoplasm of anus and anal canal (p=0.035, OR (95%CI)=1.907(1.046-3.476)) (Figure 1).

### MR analysis of eosinophil count and cancer risk based on UK Biobank

**Figure 2:**
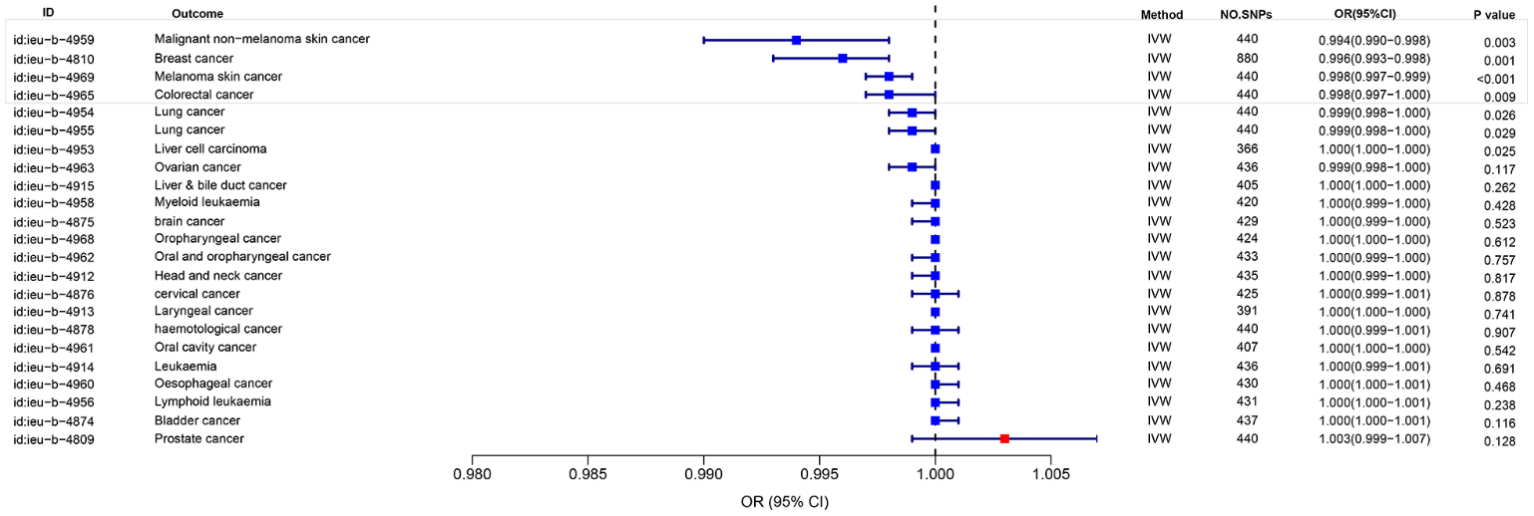
MR analysis of eosinophil count and cancer risk based on UK Biobank.

In order to investigate the correlation between eosinophil count and cancer risk by utilizing the eosinophil count with ID ieu-b-33 as the exposure data and examining 23 types of cancer in the UK Biobank as the outcome. The findings revealed an inverse relationship between eosinophil count and the risk of Melanoma skin cancer, Breast cancer, Malignant non-melanoma skin cancer, Colorectal cancer, Liver cell carcinoma, Lung cancer. And the P-values were all < 0.05. OR were all ≤1, and the maximum value of 95% CI were all ≤1 (Figure 2).

### MR analysis of eosinophil percentage and cancer risk based on FinnGen database

**Figure 3:**
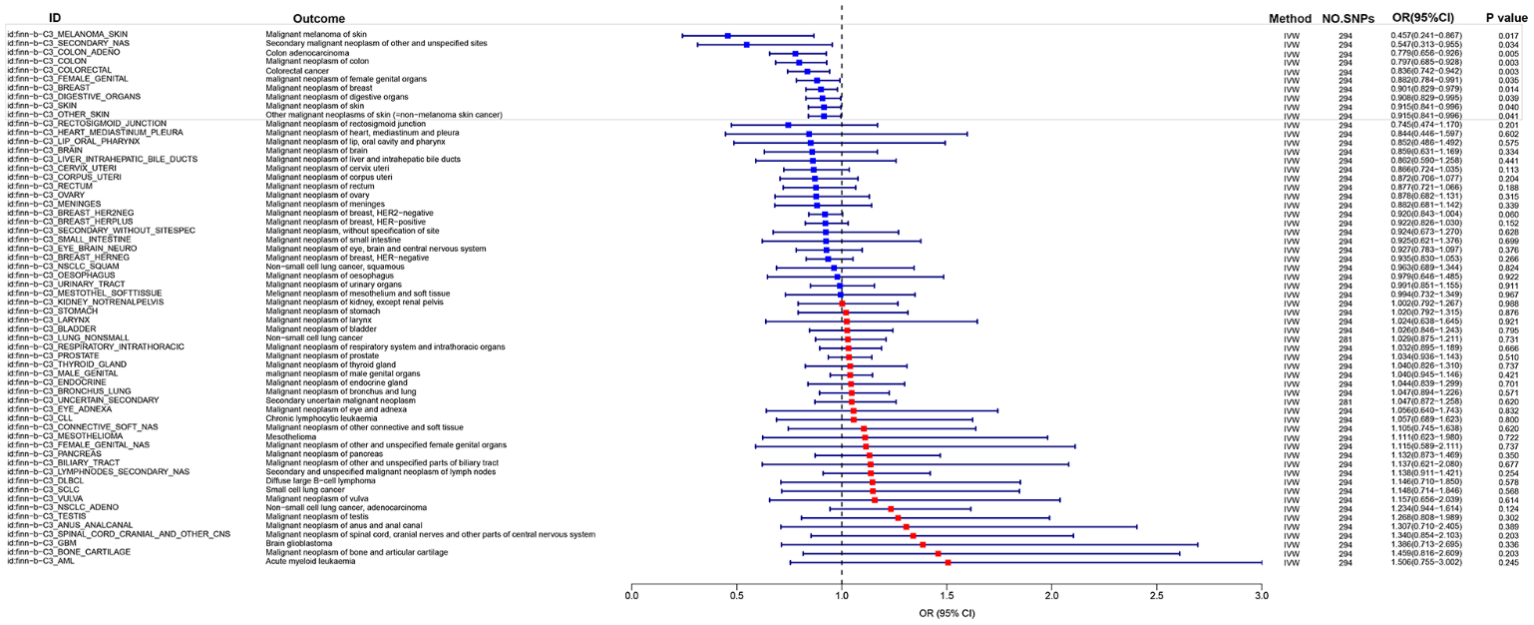
MR analysis of eosinophil percentage and cancer risk based on FinnGen database.

In order to investigate the correlation between Eosinophil percentage and cancer risk by utilizing the Eosinophil percentage with ID ukb-d-30210_irnt as the exposure data and examining 60 types of cancer in the FinnGen database as the outcome. The findings revealed an inverse relationship between Eosinophil percentage and the risk of Malignant neoplasm, Colorectal cancer, Malignant neoplasm of colon, Colon adenocarcinoma, Malignant neoplasm of breast, Malignant melanoma of skin Secondary malignant neoplasm of other and unspecified sites, malignant neoplasm of female genital organs, Malignant neoplasm of digestive organs, Malignant neoplasm of skin, Other malignant neoplasms of skin (=non-melanoma skin cancer). And the P-values were all < 0.05 OR were all < 1, and the maximum value of 95% CI were all < 1 (Figure 3).

### MR analysis of eosinophil percentage and cancer risk based on UK Biobank

**Figure 4:**
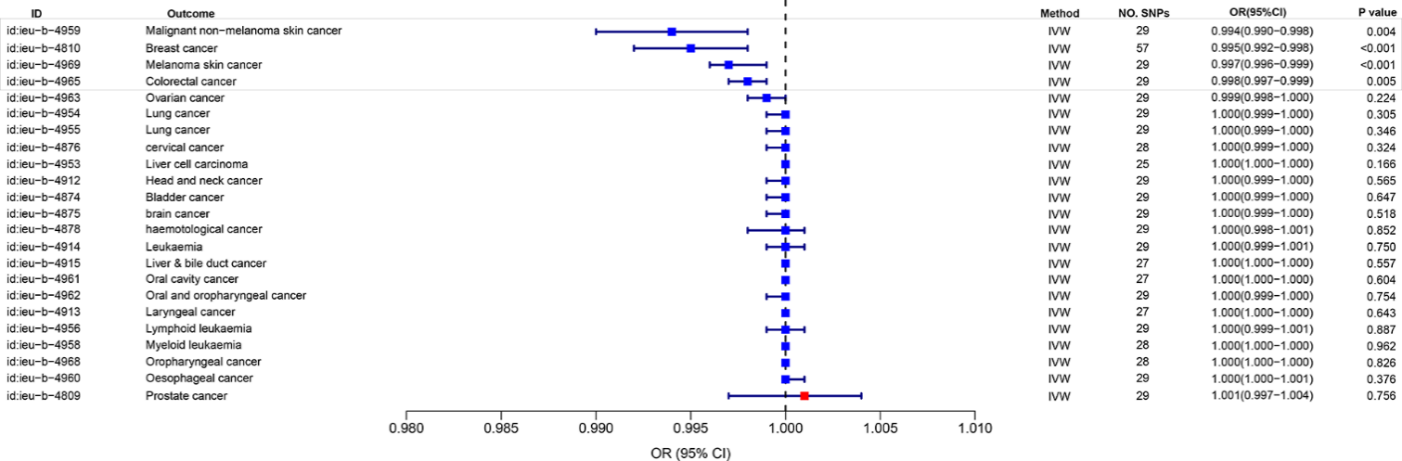
MR analysis of eosinophil percentage and cancer risk based on UK Biobank.

In order to investigate the correlation between eosinophil count and cancer risk by utilizing the eosinophil count with ID ukb-d-30210_irnt as the exposure data and examining 23 types of cancer in the UK Biobank as the outcome. The findings revealed an inverse relationship between eosinophil count and the risk of Melanoma skin cancer, Breast cancer, Malignant non-melanoma skin cancer, Colorectal cancer. And the P-values were all < 0.05. OR were all < 1, and the maximum value of 95% CI were all < 1 (Figure 4).

### MR analysis of eosinophil percentage and eosinophil count with risk of colorectal cancer and skin malignancies

In order to enhance the accuracy of this causal relationship, we further eliminated SNP confounders associated with inflammatory bowel disease, Crohn’s disease, BMR, BMI, diabetes mellitus, high cholesterol, etc. Furthermore, we employed the PRESSO test to identify and exclude outliers, and reinforced the criteria for selecting instrumental variables (p = 5e-9, R2 = 0.0001). Moreover, sensitivity analysis encompassing heterogeneity, pleiotropy, and directionality was conducted.

The analysis revealed that the percentage of eosinophils exhibited an inverse association with the risk of Malignant non-melanoma skin cancer in the UK Biobank (P=0.001, OR (95%CI) = 0.993 (0.989-0.997)) (Table 1). Additionally, eosinophil count was inversely associated with the risk of both colorectal cancer and skin malignancies from the FinnGen database and the UK Biobank (P < 0.05) (Table 1). Consistent analysis results across multiple datasets. However, there was heterogeneity in eosinophil count and the risk of certain cutaneous malignancies, with a significance level of P < 0.05. The results of the pleiotropy and heterogeneity tests can be found in the Supplementary Table 1.

**Table 1:** MR analysis of eosinophil percentage and eosinophil cell count with risk of colorectal cancer and skin malignancies.

**Exposure**	**id.outcome**	**Outcome**	**Method**	**NO.SNPs**	**OR (95%CI)**	**P-value**
**Eosinophil cell count**	finn-b-C3_COLORECTAL	Colorectal cancer	IVW	205	0.851(0.725-0.998)	**0.047**
	finn-b-C3_SKIN	Malignant neoplasm of skin	IVW (multiplicative random effects)	204	0.889(0.801-0.987)	**0.028**
	finn-b-C3_OTHER_SKIN	Other malignant neoplasms of skin (=non-melanoma skin cancer)	IVW (multiplicative random effects)	204	0.889(0.801-0.987)	**0.028**
	ieu-b-4965	Colorectal cancer	IVW	212	0.998(0.997-1.000)	**0.033**
	ieu-b-4969	Melanoma skin cancer	IVW	211	0.998(0.997-1.000)	**0.008**
	ieu-b-4959	Malignant non-melanoma skin cancer	IVW (multiplicative random effects)	203	0.994(0.989-0.998)	**0.004**
**Eosinophil percentage**	finn-b-C3_COLORECTAL	Colorectal cancer	IVW	148	0.887(0.757-1.040)	0.140
	finn-b-C3_SKIN	Malignant neoplasm of skin	IVW (multiplicative random effects)	148	0.932(0.844-1.030)	0.169
	finn-b-C3_OTHER_SKIN	Other malignant neoplasms of skin (=non-melanoma skin cancer)	IVW	147	0.941(0.855-1.036)	0.214
	ieu-b-4965	Colorectal cancer	IVW	147	0.999(0.997-1.001)	0.171
	ieu-b-4969	Melanoma skin cancer	IVW (multiplicative random effects)	147	0.999(0.997-1.000)	0.115
	ieu-b-4959	Malignant non-melanoma skin cancer	IVW	143	0.993(0.989-0.997)	0.001

### MR analysis of colorectal cancer and skin malignancies with risk of eosinophil count

The MR analysis performed on both cohorts, investigating the correlation between colorectal cancer, skin malignancies, and eosinophil count, yielded no statistically significant causal relationship, as evidenced by a p-value exceeding 0.05 (Table 2).

**Table 2:** MR analysis of colorectal cancer and skin malignancies with risk of eosinophil count.

**Id. Exposure**	**Exposure**	**id.outcome**	**Outcome**	**Method**	**NO.SNPs**	**OR (95%CI)**	**P-value**
**finn-b-C3_COLORECTAL**	Colorectal cancer	ieu-b-33	eosinophil cell count	IVW	2	0.9976(0.9824-1.0131)	0.762
**finn-b-C3_SKIN**	Malignant neoplasm of skin	ieu-b-33	eosinophil cell count	IVW	20	1.0039(0.9970-1.0109)	0.269
**finn-b-C3_OTHER_SKIN**	Other malignant neoplasms of skin (=non-melanoma skin cancer)	ieu-b-33	eosinophil cell count	IVW	21	1.0046(0.9977-1.0114)	0.192
**ieu-b-4965**	Colorectal cancer	ieu-b-33	eosinophil cell count	IVW	7	1.3853(0.5766-3.3282)	0.466
**ieu-b-4969**	Melanoma skin cancer	ieu-b-33	eosinophil cell count	IVW	9	1.0268(0.5055-2.0860)	0.942
**ieu-b-4959**	Malignant non-melanoma skin cancer	ieu-b-33	eosinophil cell count	IVW (multiplicative random effects)	38	0.9861(0.8295-1.1723)	0.874

### MR analysis of asthma and risk of colorectal cancer and skin malignancies

**Figure 5:**
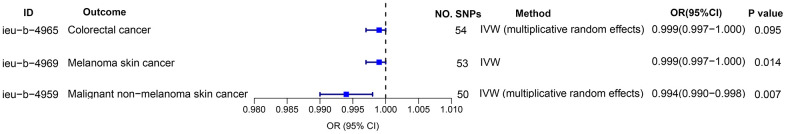
MR analysis of asthma and risk of colorectal cancer and skin malignancies.

The results of the Mendelian randomization analysis revealed an inverse association between asthma and the risk of colorectal cancer and skin malignancies. The statistical analysis indicated that the P-values for the relationship between asthma and the risk of skin malignancies were all < 0.05, suggesting a significant association. Conversely, the P-values for the association between asthma and the risk of colorectal cancer were greater than 0.05, but close to 0.05. OR=0.999, 95%CI (0.997-1.000), it also proves to some extent that there is a negative correlation between asthma and the risk of colorectal cancer (Figure 5). The results of the pleiotropy and heterogeneity tests can be found in the Supplementary Table 2.

### Multivariate MR analysis of asthma, eosinophil count, and risk of colorectal cancer and skin malignancies

The results of the multivariate MR analysis revealed a significant negative correlation between the eosinophil count and the risk of both colorectal cancer and skin malignancy (P < 0.05) (Table 3). Conversely, the P-values for the association between asthma and the risk of colorectal cancer and skin malignancy were all above 0.05 (Table 3). The results of the pleiotropy and heterogeneity tests can be found in the Supplementary Table 3.

**Table 3:** Multivariate MR analysis of asthma, eosinophil count, and risk of colorectal cancer and skin malignancies.

**Id. Exposure**	**Exposure**	**id.outcome**	**Outcome**	**OR (95%CI)**	**P-value**
**ebi-a-GCST90014325**	Asthma	ieu-b-4965	Colorectal cancer	1.000(0.999-1.002)	0.6648
**ieu-b-33**	eosinophil cell count	ieu-b-4965	Colorectal cancer	0.998(0.997-1.000)	**0.0258**
**ebi-a-GCST90014325**	Asthma	ieu-b-4969	Melanoma skin cancer	1.001(0.999-1.002)	0.4068
**ieu-b-33**	eosinophil cell count	ieu-b-4969	Melanoma skin cancer	0.998(0.996-0.999)	**0.0002**
**ebi-a-GCST90014325**	Asthma	ieu-b-4959	Malignant non-melanoma skin cancer	1.001(0.996-1.006)	0.8067
**ieu-b-33**	eosinophil cell count	ieu-b-4959	Malignant non-melanoma skin cancer	0.995(0.990-0.999)	**0.0233**

### MR analysis of eosinophils mediated by gene IL-4, IL-5, IL-13, IL-4R, and IL-5RA and risk of colorectal cancer and skin malignancies based on UK Biobank

**Figure 6:**
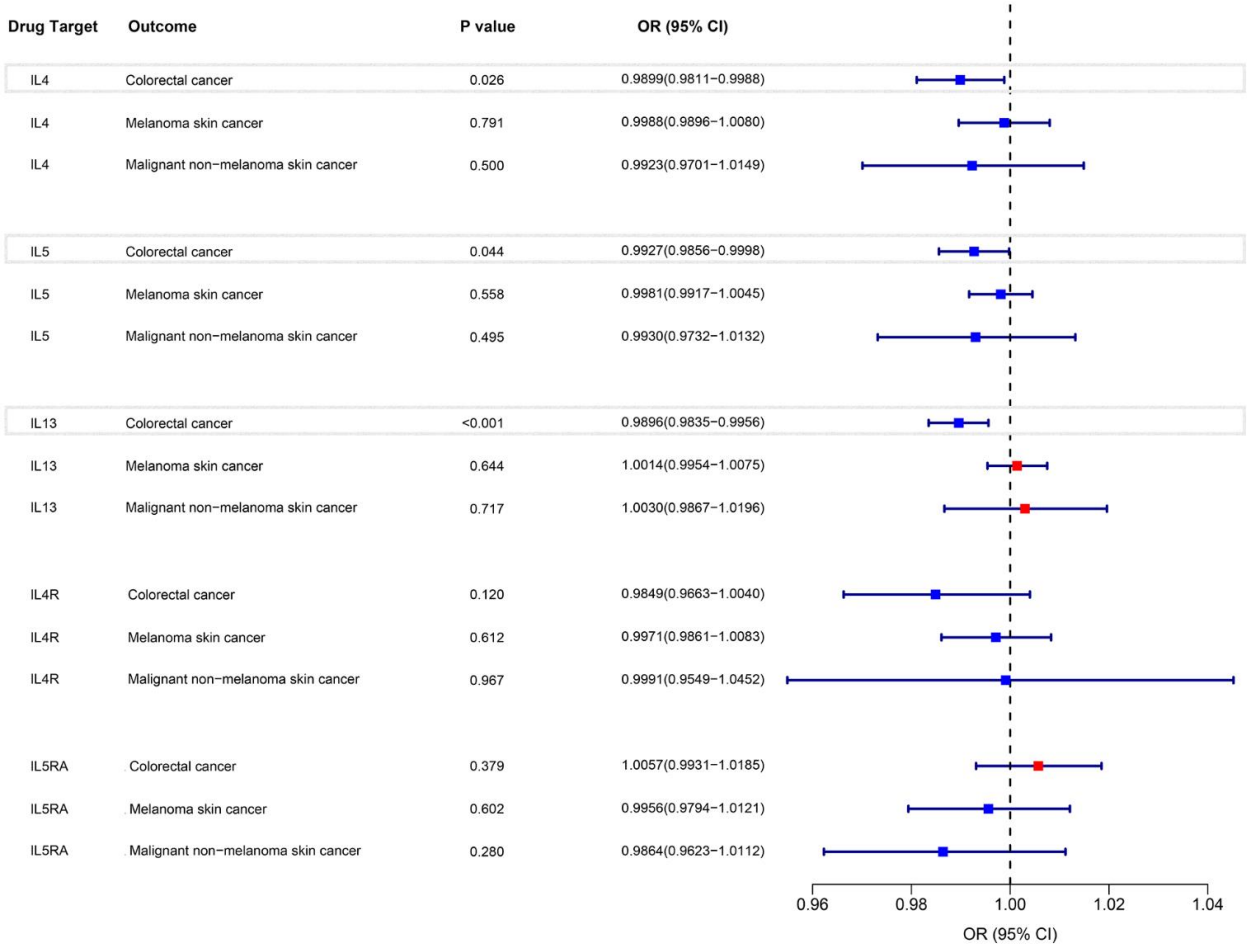
IVW MR analysis between eosinophils mediated by gene IL-3, IL-4, IL-5, IL-4R, and IL-5RA and colorectal cancer and skin malignancies outcomes.

The IVW method was employed to examine the association between eosinophils regulated by gene IL-4, IL-5, IL-13, IL-4R, and IL-5RA and the risk of colorectal cancer and skin malignancies. The analysis revealed a negative correlation between eosinophils regulated by IL-4, IL-5, and IL-13 and the risk of colorectal cancer (P < 0.05). OR were all < 1, and the maximum value of 95% CI were all < 1 (Figure 6). However, there was no significant association between eosinophils regulated by IL4R, IL5RA, and the risk of colorectal cancer (P > 0.05) (Figure 6). Nevertheless, a tendency towards an inverse association was observed between IL4R-regulated eosinophils and colorectal cancer risk (Figure 6). Furthermore, eosinophils regulated by IL-4, IL-5, IL-13, IL-4R, and IL-5RA were not found to be associated with the risk of skin malignancies (Figure 6). The results of the pleiotropy and heterogeneity tests can be found in the Supplementary Table 4.

### MR analysis of eosinophils mediated by gene IL-4, IL-5, IL-13, IL-4R, and IL-5RA and risk of colorectal cancer based on FinnGen database

**Figure 7:**
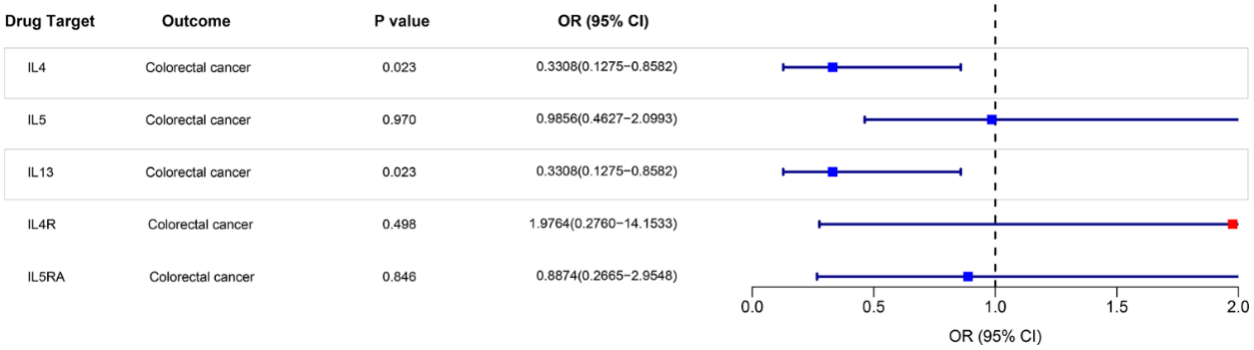
IVW MR analysis between eosinophils mediated by gene IL-3, IL-4, IL-5, IL-4R, and IL-5RA and colorectal cancer outcomes.

The analysis revealed a negative correlation between eosinophils regulated by IL-4, and IL-13 and the risk of colorectal cancer (P < 0.05). OR were all < 1, and the maximum value of 95% CI were all < 1 (Figure 7). However, there was no significant association between eosinophils regulated by IL-5, IL4R, IL5RA, and the risk of colorectal cancer (P > 0.05) (Figure 7). The results of the pleiotropy and heterogeneity tests can be found in the Supplementary Table 5.

## DISCUSSION

Eosinophils constitute a crucial element within the tumor immune microenvironment, exhibiting dual functionality. Firstly, they possess the capability to eliminate tumor cells via direct or indirect mechanisms. Conversely, they also release soluble mediators that facilitate angiogenesis, matrix remodeling, and ultimately foster tumor progression [2]. Consequently, the association between eosinophil count in the bloodstream and cancer susceptibility remains unclear due to the absence of comprehensive pan-cancer investigations. To address this knowledge gap, Mendelian randomization, a reliable predictor of causality and a safeguard against confounding factors [7, 8], can be employed. In particular, the instrumental variable weighted (IVW) method serves as the primary analytical approach within Mendelian randomization studies [9].

Initially, the IVW method was employed to examine the correlation between eosinophil count and eosinophil percentage in relation to various cancer risks using data from the European FinnGen database and the UK Biobank. Analysis of tumor data from both cohorts revealed a negative association between eosinophil count and eosinophil percentage with the risk of Colorectal cancer, Melanoma skin cancer, and Malignant non-melanoma skin cancer. Subsequently, in order to enhance the accuracy of this causal relationship, we further eliminated SNP confounders associated with inflammatory bowel disease, Crohn’s disease, BMR, BMI, diabetes mellitus, high cholesterol, etc. Furthermore, we employed the PRESSO test to identify and exclude outliers, and reinforced the criteria for selecting instrumental variables (p = 5e-9, R2 = 0.0001). Moreover, sensitivity analysis encompassing heterogeneity, and pleiotropy was conducted. The findings from the Mendelian randomization analysis indicate that there is an association between eosinophil percentage and the risk of malignant non-melanoma skin cancer, only in the UK Biobank oncology data (P < 0.05). However, the heterogeneity detection P-value is also less than 0.05. No statistically significant relationship was observed between eosinophil percentage and the risk of other colorectal cancers or skin malignancies.

The analyses conducted on the eosinophil count and its association with the risk of colorectal cancer and skin malignancies revealed statistically significant results, with P-values below 0.05. However, it is important to note that heterogeneity was observed in the relationship between eosinophil count and the risk of skin malignancies from the FinnGen database and malignant non-melanoma skin cancer from the UK Biobank. Notably, the eosinophil count exhibited an inverse association with the risk of colorectal cancer in both cohorts, with statistical significance (P < 0.05), and no evidence of heterogeneity or pleiotropy. And reverse Mendelian randomization analysis shows that there is no reverse causal relationship between them. Consequently, it can be concluded that eosinophil count exhibits an inverse relationship with the risk of colorectal cancer, supported by robust statistical evidence. However, it is worth noting that the relationship between eosinophil count and the risk of skin malignancy may be influenced by additional factors.

Several studies have additionally demonstrated the anti-cancer properties of eosinophils against melanoma [10] and colorectal cancer [11]. Specifically, eosinophils induced by human embryonic stem cells have been observed to impede the proliferation of HCT116 colon cancer cells in immunodeficient mice, leading to an extended median survival time and suppression of tumor formation. This phenomenon is believed to be attributed to the release of EPX, EDN, and granzyme A by eosinophils [12]. Furthermore, the presence of both blood eosinophils [13] and tumor-infiltrating eosinophils [14] has been correlated with the prognosis of colorectal cancer.

Epidemiological research has demonstrated a significant association between allergies and cancer. Allergic conditions have the potential to impede tumorigenesis by enhancing immune surveillance, yet they may also facilitate tumor progression through the inflammatory response triggered by allergies [15]. Among various allergic diseases, asthma stands as a prevalent example [16]. In this study, we conducted an analysis to explore the correlation between asthma and the susceptibility to colorectal cancer and skin malignant tumors. Our findings indicate a negative association between asthma and the risk of colorectal cancer and skin malignant tumors (with a p-value for colorectal cancer risk exceeding 0.05, but approaching 0.05).

Patients with allergies frequently exhibit eosinophilia [17]. A subsequent Mendelian randomization analysis investigating the relationship between asthma, eosinophil count, and susceptibility to colorectal cancer and skin malignancy revealed that asthma per se did not significantly impact the susceptibility to these malignancies (p > 0.05). However, eosinophils played a significant mediating role in the inverse association observed between asthma and the risk of colorectal cancer and skin malignancies (p < 0.05).

A number of recently approved biologic therapies have been employed to treat diseases characterized by eosinophilia, focusing on the regulation factors of eosinophils, namely interleukin (IL) 4 or IL 5 and their receptors, IL 13 [1, 18, 19]. However, the potential impact of these drugs on the susceptibility of related tumors remains unexplored. Through the use of MR analysis, our study revealed an inverse association between IL-4, and IL-13-regulated eosinophils and the risk of colorectal cancer in both cohorts. Consequently, it is plausible that drugs targeting IL-4, and IL-13 may elevate the risk of developing colorectal cancer.

Collectively, our findings lead us to deduce that there exists an inverse correlation between eosinophils count and the risk of colorectal cancer and skin malignancies. Additionally, eosinophils serve as a mediating factor in asthma, thereby diminishing susceptibility to colorectal cancer and skin malignancies. The statistical data provide the most robust evidence for the negative association between eosinophils count and the risk of colorectal cancer. Moreover, some medications that modulate eosinophils may heighten the risk of colorectal cancer. Consequently, caution should be exercised in the future regarding the utilization of such drugs, taking into consideration the potential risk of colorectal cancer.

This study primarily constitutes a Mendelian randomization (MR) analysis utilizing genome-wide association study (GWAS) data, which has some limitations. Subsequent investigations should aim to validate the precise factors contributing to the inverse correlation between eosinophils and colorectal cancer risk through fundamental experiments. Furthermore, it is imperative to elucidate the mechanisms by which drugs modulate eosinophils to mitigate the risk of colorectal cancer. Additionally, considering the stringent criteria employed in our study, eosinophils count or eosinophils percentage may potentially exhibit associations with other cancer risks. Even so, it is worth noting that the inverse association between eosinophils count and the risk of colorectal cancer has been consistently observed in colorectal cancer data from sources such as the UK Biobank and FinnGen database. This suggests that the relationship between eosinophils count and colorectal cancer risk is relatively robust.

## MATERIALS AND METHODS

### Study design

**Figure 8:**
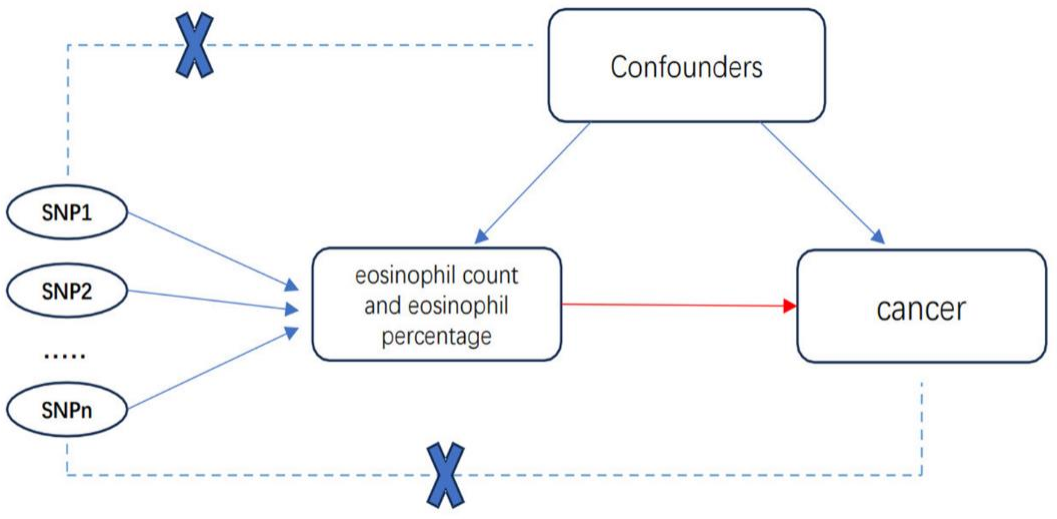
Basic principles and assumptions of MR.

The design is as follows:

1. MR is a technique that utilizes genetic variation as an instrumental variable to estimate the causal association between exposure and outcomes. The term “exposure” typically denotes a presumed causal risk factor, with diseases commonly serving as the outcomes of interest. The detailed principle and statistical method of MR are described in detail by Eleanor Sanderson et al. [20]. In our investigation, we employed eosinophil count and percentage as the exposure variables, with cancers as the designated outcomes, as depicted in the accompanying figure (Figure 8). The specific steps involved in instrumental variable selection will be detailed in the subsequent methods section.
2. We analyzed eosinophil count and eosinophil percentage in relation to cancer risk from the UK Biobank and FinnGen databases using inverse-variance weighted mendelian randomization (IVW-MR) analysis (P < 5 × 10 – 8, r2 = 0.001, kb = 10000).
3. Subsequent intersection of risk-related tumors from both cohorts.
4. Sensitization analysis of tumors in the intersection was further performed, and the restriction of instrumental variables was strengthened (P < 5 × 10 – 9, r2 = 0.0001, kb = 10000), and confounding factors, outliers, and SNPs related to outcome (P < 5 × 10 – 5) were also removed. Only the eosinophil count was found to be associated with related cancer risk.
5. Reverse MR analysis (P < 5 × 10-8, R2 = 0.001, kb = 10000) for reverse causality.
6. Univariate and multivariate MR analysis (P < 5 × 10 – 8, r2 = 0.001, kb = 10000) of the association between asthma and the risk of related cancers to identify the mediating role of eosinophils.
7. MR analysis (P < 5 × 10 – 8, r2 = 0.01, kb = 100) of the relationship between drug-regulated eosinophils and the risk of related tumors to determine whether the drugs affect the risk of related tumors.

**Figure 9:**
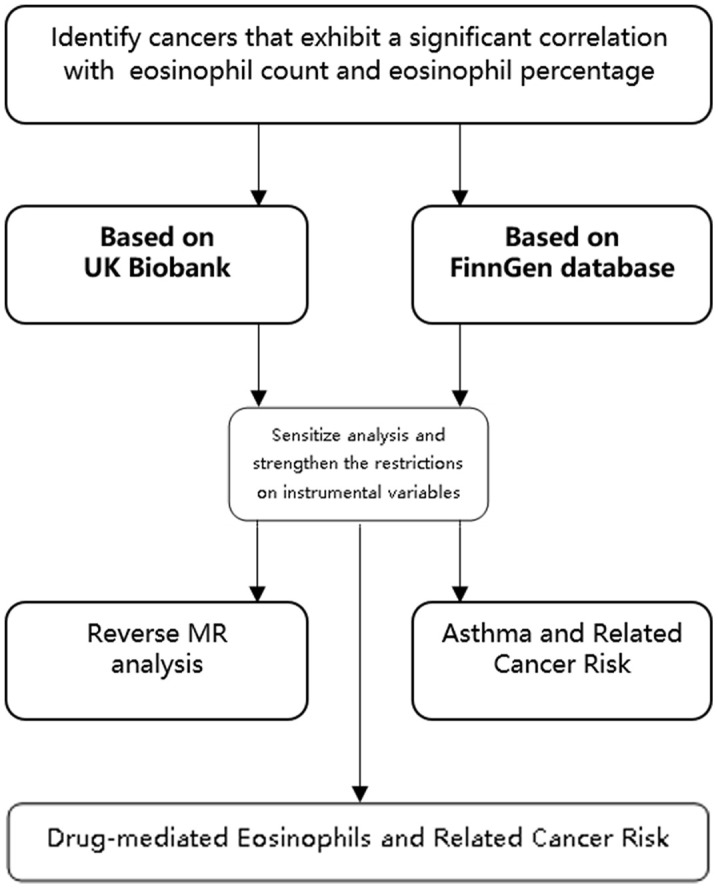
Study design.

The study design is shown in Figure 9.

### Data sources

All the GWAS data mentioned in this article were obtained from the IEU GWAS database (https://gwas.mrcieu.ac.uk/). Specifically, a total of 60 tumor GWAS data from the FinnGen database and 23 tumor GWAS data from the UK Biobank were downloaded. Additionally, the eosinophil cell count data with ID ieu-b-33 and the eosinophil percentage data with ID ukb-d-30210_irnt were utilized. Furthermore, asthma data with ID ebi-a-GCST90014325 is also accessible, which were limited to the European population. Detailed information on each tumor can be found in Supplementary Table 6.

### Selection of instrumental variables

In the majority of the Mendelian randomization (MR) analyses conducted in this study, single nucleotide polymorphisms (SNPs) that showed a statistically significant association with the relevant exposure at the genome-wide level of significance (P-value < 5 × 10 – 8) and demonstrated no linkage disequilibrium (LD) with other SNPs (r2<0.001 within a clumping window of 10000 kilobase (kb)) were utilized as instrumental variables for these exposures.

However, in the MR analysis investigating the relationship between eosinophil count and eosinophil percentage and the risk of colorectal cancer and skin malignancy, the selection criteria for instrumental variables were strengthened. SNPs that exhibited a significant association with the relevant exposure at the genome-wide level of significance (P-value < 5 × 10 – 9) and demonstrated no linkage disequilibrium (LD) with other SNPs (r2<0.0001 within a clumping window of 10000 kb) were employed as instruments for these exposures.

For the purpose of selecting instrumental variables for drug-regulated eosinophils, we employed single-nucleotide polymorphisms situated within a 100 kilobase range of relevant genes and exhibiting a significant association with Eosinophil count at a genome-wide significance level of P < 5× 10 – 8 as instruments. These instruments were also subjected to clumping based on a linkage disequilibrium threshold of r2 < 0.01.

### Find and remove confounders and outliers

Confounding factors associated with the outcome were identified using PhenoScanner V2 [21]. Outliers that may influence the causal effects detected by the MR-PRESSO global test [22] were identified. Additionally, the single nucleotide polymorphism (SNP) linked to the outcome (P < 5 × 10 – 5) was eliminated.

### Sensitivity analysis

The F-statistic was employed to evaluate the efficacy of SNPs as instruments, and SNPs with an F-statistic exceeding 10 were incorporated to mitigate the potential influence of weak instrument bias. In addition, to evaluate potential heterogeneity among causal effects of different variants, the χ2 Q test was employed, and a P-value of less than 0.05 was regarded as significant heterogeneity. The MR-Egger intercept analysis evaluated the horizontal pleiotropy, which means IVs affect both exposure and outcome through a pathway not mediated by causal effect [23]. The no evidence of horizontal pleiotropy (MR-Egger intercept < 0.01, p-value > 0.05).

### Statistical analysis

All MR analyses were performed using R (version 4.3.0) with packages “TwoSampleMR”, “MendelR”, and “MRPRESSO”. The inverse-variance weighted (IVW) method was used as our main MR method to detect exposure to outcome [9]. We adopted the random-effects IVW model if heterogeneity existed, otherwise fx-effects IVW model was used. P < 0.05 was statistically significant.

### Availability of data and materials

This study is an analysis of existing data in the IEU GWAS database, which can be downloaded from the website described in this article.

## Supplementary Material

Supplementary Table 1

Supplementary Table 2

Supplementary Table 3

Supplementary Table 4

Supplementary Table 5

Supplementary Table 6
